# Artificial Intelligence-Driven Diagnosis of Pancreatic Cancer

**DOI:** 10.3390/cancers14215382

**Published:** 2022-10-31

**Authors:** Bahrudeen Shahul Hameed, Uma Maheswari Krishnan

**Affiliations:** 1 Centre for Nanotechnology & Advanced Biomaterials (CeNTAB), Shanmugha Arts, Science, Technology and Research Academy, Deemed University, Thanjavur 613401, India; 2 School of Chemical & Biotechnology (SCBT), Shanmugha Arts, Science, Technology and Research Academy, Deemed University, Thanjavur 613401, India; 3 School of Arts, Sciences, Humanities & Education (SASHE), Shanmugha Arts, Science, Technology and Research Academy, Deemed University, Thanjavur 613401, India

**Keywords:** pancreatic cancer, artificial intelligence, deep learning, cancer imaging, risk prediction

## Abstract

**Simple Summary:**

Pancreatic cancer poses a grave threat to mankind, due to its poor prognosis and aggressive nature. An accurate diagnosis is critical for implementing a successful treatment plan given the risk of exacerbation. The diagnosis of pancreatic cancer relies on medical imaging, which provides inaccurate information about the prognosis of the patient and makes it difficult for clinicians to select the optimal treatment. Data derived from medical imaging has been integrated with artificial intelligence, an emerging technology, to facilitate clinical decision making. This review explores the implementation of artificial intelligence for various imaging modalities to obtain a precise cancer diagnosis.

**Abstract:**

Pancreatic cancer is among the most challenging forms of cancer to treat, owing to its late diagnosis and aggressive nature that reduces the survival rate drastically. Pancreatic cancer diagnosis has been primarily based on imaging, but the current state-of-the-art imaging provides a poor prognosis, thus limiting clinicians’ treatment options. The advancement of a cancer diagnosis has been enhanced through the integration of artificial intelligence and imaging modalities to make better clinical decisions. In this review, we examine how AI models can improve the diagnosis of pancreatic cancer using different imaging modalities along with a discussion on the emerging trends in an AI-driven diagnosis, based on cytopathology and serological markers. Ethical concerns regarding the use of these tools have also been discussed.

## 1. Introduction

Pancreatic cancer (PC) is among the most fatal and invasive tumors of the digestive system [1]. It has been referred to as the ‘king of cancer’, due to its aggressiveness, invasiveness and rapid metastasis, poor survival, and poor prognosis [2]. Recent decades have witnessed a surge in the incidence of pancreatic cancer across the globe that has been largely linked to ageing, alcohol consumption, smoking, sedentary lifestyle, obesity, chronic pancreatitis, diabetes, hereditary factors, long-term exposure to air and water pollutants, unhealthy lifestyle, and diet [1,3,4]. Surgery has been the main therapeutic intervention for these patients. However, several factors, including the absence of specific clinical manifestations and molecular markers, have resulted in the detection of the disease only at advanced stages, thereby making surgical options ineffective. Therefore, an early diagnosis and accurate stratification of pancreatic cancer stages are important for improved therapeutic outcomes. Pancreatic cancer diagnosis is challenging because the pancreas is a deep-seated retro-peritoneal organ with complex surrounding structures. The highly vascularized environment surrounding the pancreas facilitates rapid metastasis of the cancer that makes pancreatic cancer highly aggressive. The common symptoms of pancreatic cancer include abdominal pain, changes in the consistency of faeces, nausea, bloated body, co-morbidities, such as diabetes and jaundice, abnormal liver function parameters, loss of weight, etc. [5]. These symptoms usually become prominent only during the advanced stage of the disease and are often missed during the early stages. Further, serological markers for pancreatic cancer, such as CA-19-9 (Carbohydrate antigen), are not highly specific and indicate only the advanced stage of the disease, thereby increasing the mortality risk of the affected individual. Several imaging tools, including magnetic resonance imaging (MRI), computed tomography (CT), endoscopic ultrasound (EUS), etc., have also been explored for the diagnosis of pancreatic cancer. Due to rapid advances in recent years, imaging technology has emerged in the forefront for the diagnosis, staging, and prognosis of pancreatic cancer [6]. However, distinction of a cancerous lesion from other pancreatic disorders, such as pancreatitis, a chronic inflammation of the pancreas, remains a major roadblock in the accurate and early diagnosis of pancreatic cancer. Despite the existence of advanced imaging equipment, confirmation of pancreatic cancer is confirmed through biopsy after imaging. Not only is this time-consuming, but it also increases the probability of mortality in the affected individual, due to the inordinate delay. A study had reported that nearly 90% of the misdiagnosis of pancreatic cancer was due to the inability to identify the vascular invasion and the difficulty in spotting the underlying tumour mass, due to the inflammation [7]. Table 1 lists some of the common imaging techniques used for the clinical diagnosis of pancreatic cancer, along with their merits and limitations.

Several approaches to improve the sensitivity and prediction accuracy of these imaging techniques have been reported in the literature. These include the use of image contrast agents to improve the resolution and sensitivity and the use of image processing software for a better diagnostic accuracy. In recent years, the emergence of artificial intelligence and deep learning has transformed the landscape of an image-driven diagnosis of pancreatic cancer with a dramatic improvement in the prediction accuracy. The various attempts to integrate artificial intelligence for the diagnosis of pancreatic cancer are discussed in the following sections.

## 2. Artificial Intelligence for Diagnostic Applications

The advances in computer technology, witnessed in the recent decades, coupled with the development of effective image processing strategies, have ushered in a new era of ‘digital medicine’. As a result, clinical personnel can avoid the laborious medical image analysis performed manually, thus saving time as well as overcome errors in diagnosis arising, due to the differences in expertise and clinical exposure [8]. The 21st century has witnessed the widespread use of artificial intelligence (AI) that employs computer programs to perform tasks associated with human intelligence, such as learning and problem-solving. The phrase ‘artificial intelligence’ was first coined by John McCarthy in the mid-1950s, and has since evolved from a set of ‘if-then’ commands to more complex algorithms that mimic the human brain in some aspects [9,10]. The application of AI tools has resulted in the emergence of a new field of clinical diagnosis, namely, precision oncology that uses a large volume of data from genomics, proteomics, and metabolomics [11]. AI-based cancer diagnosis is mainly driven by machine learning (ML) and deep learning (DL) techniques. Machine learning uses computational methods to analyse large volumes of data and identify patterns for prediction [12]. ML can be supervised where it uses data from previous trials/measurements for the identification of patterns or trends for making predictions. Thus, for a pancreatic cancer diagnosis, CT or PET scans, ultrasonographs, and MRI data can be used to train the system to identify abnormalities that can be classified as pancreatic cancer. The prediction accuracy can be better if large numbers of dataset are used for the training. Different mathematical models and algorithms can be iteratively used during the training period to identify the most efficient model, the accuracy of which can be validated using a testing dataset. The advantage of such supervised ML models is that they can extract meaningful features and identify patterns or subtle changes that could be missed by human personnel, due to oversight or exhaustion. Hence, the prediction accuracy of ML for a cancer diagnosis is higher. ML can also be unsupervised where it can discern patterns and trends from unclassified data. However, the accuracy of the prediction is slightly compromised when compared to the supervised models [13]. The 3D reconstruction of images has also been realized by the ML models for a superior diagnostic accuracy [6].

Another type of ML that is yet to be applied for cancer diagnosis, is reinforcement learning where the algorithm uses the data to understand and respond to the environment predominantly by a trial-and-error process [14]. In other words, reinforcement learning is an advanced concept that could also facilitate decision-making, in addition to prediction [15]. Thus, apart from a diagnosis of pancreatic cancer, reinforcement learning could be used to alert clinicians in remote locations or trigger actuators for releasing a therapeutic agent. These concepts, though attractive, are yet to be realized, but could very well represent the diagnostic technology of the future. Deep learning is another sub-type of AI that uses large data sets and complex algorithms that mimic the human brain to enable prediction, forecasting, and decision-making [16,17]. Most of the DL is supervised and uses data for training for the decision-making process, unlike reinforcement learning that is a dynamic process which relies on a trial-and-error method for the same. Both DL and reinforcement learning are advanced concepts that require a longer duration for training and testing [18]. DL employs convolutional neural networks (CNNs) and artificial neural networks (ANNs) extensively for decision-making [19].

**Figure 1:**
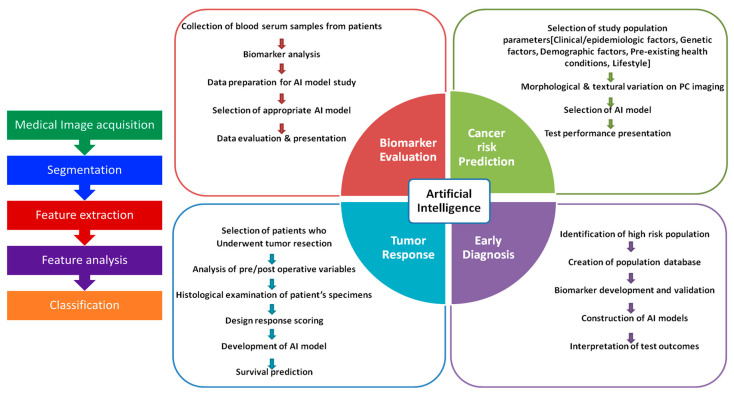
Work-flow of the stages during the training of the ML models for the diagnosis of cancer lesions.

A plethora of supervised and unsupervised ML and DL models continue to be developed and explored for improving the accuracy of a pancreatic cancer diagnosis at the early stage which could be invaluable in enhancing the survival of the affected individual [20]. The complexity of the algorithms will reflect the type of functions they can perform ranging from feature extraction, simple clustering or segregation of data, classification of data, prediction, forecasting, and decision-making [21]. Algorithms such as Naive–Bayes, support vector machine, linear regression analysis, ensemble methods, decision tree, K-mode, hidden Markov model, hierarchical, Gaussian mixture, and neural networks have all been explored with different imaging data sets for distinguishing cancerous tissue from non-cancerous tissues [22]. The work flow in the detection of cancer using ML is depicted in Figure 1.

The classification of images for diagnosis using various AI models can be broadly divided into one-stage and two-stage methods. The one-stage method segments the medical image into grids and applies the model for classification while the two-stage method demarcates several candidate zones that are used for classification during the training. Though time-consuming, the two-stage object method identifies and screens regions of interest resulting in more accurate predictions. Region-based convolution network (R-CNN), Fast R-CNN, and Faster R-CNN have been employed in the two-stage method as an integrated network for discriminative feature extraction, segmentation, and classification for an improved cancer detection without compromising the spatial structures [6].

## 3. AI Models for the Diagnosis of Pancreatic Cancer

Medical imaging has been widely used for locating and diagnosing cancerous tissue in the gastrointestinal tract. Current analysis is largely dependent upon the expertise and experience of the clinician. The quality of the images also influences the diagnosis through conventional methods [23]. The field of digital pathology continues to evolve from the first generation of image processing that involved the use of image processing tools to analyse a single slide, to much more advanced second-generation tools that could scan, analyse, and store records of whole tissue samples. The current paradigm in digital pathology involves the use of AI-based algorithms to analyse images, diagnose the condition with a high accuracy, and even predict the possibility of developing the disease even before the onset of the disease [24]. The development of AI-based tools has enabled the rapid and high precision diagnosis of cancer using different medical images [25]. In the context of pancreatic cancer, AI-based diagnostic tools have been employed for risk prediction, survival prediction, and the distinction of cancer masses from other pancreatic lesions as well as for the evaluation of the response post-therapy.

Machine learning tools, such as the K-nearest neighbour (k-NN), ANN, and SVM, have been extensively investigated for their ability to extract unique signatures from medical images that could be used for the identification of abnormalities [26] in different types of digestive system cancers that also includes pancreatic cancer [27]. The k-NN algorithm, first introduced in 1967 by Cover and Hart, calculates and predicts the distance between the values of the specified features in the sample data and training data. Based on the calculated distance, the sample data is grouped with its nearest neighbour class [28]. The k-NN concept was employed by Kilicet al. [29] to identify colonic polyps using region covariance in CT-colonography images as the distinguishing features. In another report employing k-NN [30], the gray level co-occurrence matrix was employed as the classifying feature in medical images of the brain and pancreatic cancers. However, k-NN is limited by issues pertaining to local structure sensitivity and the possibility of over-fitting, leading to errors.

**Figure 2:**
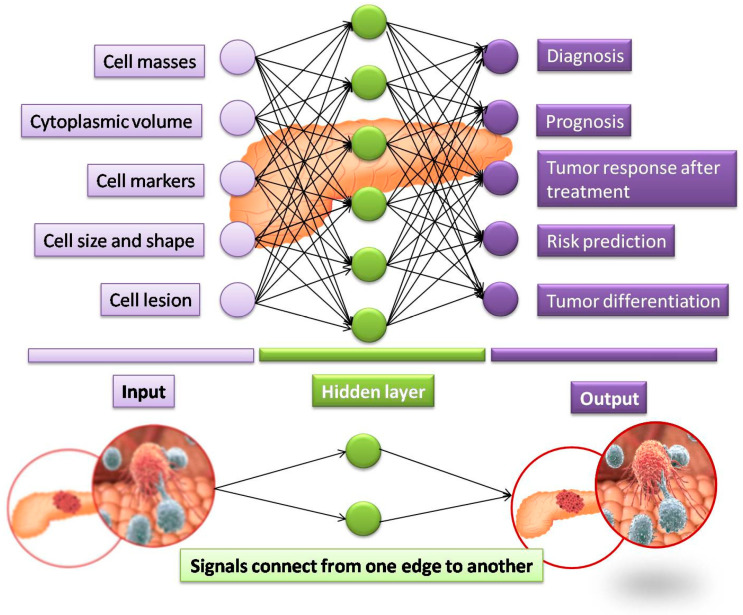
Schematic representation of the process flow in a sample ANN model for the diagnosis of pancreatic cancer.

Artificial Neural Networks (ANNs), the concept of which was first proposed in the early 1940s by McCulloch and Pitts, attempt to mimic the human neuronal network. The input layer receives the input signal that is then passed on to each of the inner hidden layers that understands and transforms them and passes it on to the next layers, until it reaches the final output layer [31], as shown in Figure 2. Unlike k-NN models that can only handle limited data, the ANN model is adaptive and can be trained using large volumes of data to become more robust and accurate. The progress in ANNs has been accelerated, due to advances in big data, affordable graphics processing units (GPUs) and the development of novel algorithms [32]. The ANN method used in diagnosing digestive cancers is the back-propagating (BP) network that was first introduced in 1986 by Rumelhart [33]. This strategy enables the error correction as the output is sent back to the inner layers if found erroneous, to refine the output parameters during the training period. This iterative process ensures the minimization of errors and the improved accuracy. In the context of a pancreatic cancer diagnosis, Săftoiu et al. [34] successfully employed ANNs to differentiate chronic pancreatitis and pancreatic adenocarcinoma, using endoscopic ultrasound images with a sensitivity of 94%. The ANN method has advantages of being able to handle large data sets and predict all types of interactions and inter-relationships between dependent and independent variables [35]. However, ANN algorithms are slow when large numbers of inputs are provided during the training period and require a large computational load, apart from adopting a black-box approach that makes it challenging for achieving accuracy in multi-layer networks [36].

To overcome some of the limitations of ANNs, Vapnik et al. [37] developed a supervised learning algorithm, in 1995, known as the support vector machine (SVM) algorithm, that defines the boundaries known as support vectors to construct a hyperplane, which is used to classify data [38]. The negative and positive boundaries and the maximum margin are defined, based upon the training set of data fed as inputs. The SVM is capable of pattern recognition and regression analysis in addition to the classification of data [39]. Zhang et al. [40] had effectively applied the SVM to identify pancreatic cancers from EUS images, by classifying textural features to achieve a detection accuracy of 99.07%. Though SVM models display a high accuracy and can work with remarkable efficiency when there is a clear demarcation of the data classes, its efficiency reduces when the size of the data set increases or when there is extensive overlap of the data. In addition, despite being memory efficient, SVM algorithms are slow, both during the training, as well as the testing phases. 

Deep learning networks exhibit superior diagnostic abilities when compared to ML models as they could extract all features rather than selected ones from the medical images, as in the case of ML. As a result, DL models are preferred for the detection of digestive cancers and image segmentation [41]. Convolutional neural networks (CNNs) are among the most extensively employed supervised DL techniques. These consist of input layers where different clusters of nodes, each for a specific feature, interact with the hidden layers that have the same weightage and bias and perform convolutional operations on these inputs. These are then pooled and transformed to give the final output [42]. A typical CNN network comprises the input, convolutional, activating, pooling, fully connected, and output layers [43]. CNNs are computationally efficient but consume lots of computational power and are slow. CNNs provide a probabilistic depiction of the complete image that can be preferably employed for the image classification, rather than the segmentation [44]. Among the various types of CNNs, U-Net algorithms that use fewer convolutional layers have also been commonly employed for the diagnosis of digestive cancers, including pancreatic cancer, by classifying and segmenting specific features in the medical images [45]. The LeNet, proposed by Lecunet al. [46] in 1989, is considered the basic structure of CNNs. Several other variants, such as AlexNet, VGGNet (visual geometry group), Inception Net, and ResNet, have been introduced, between 2012 and 2015, that vary in the number of convolutional and pooling layers employed [47]. In the context of digestive cancers, Sharma et al. [48] classified and detected necrosis in medical images of gastric carcinoma using the AlexNet architecture with a classification accuracy of 69.9% and a detection accuracy of 81%. Colonic polyps were automatically detected by Shin et al. [49] from colonoscopy images using the Inception-Resnet network. Long et al. [50] proposed a fully convolutional network (FCN) model, in 2015, for the semantic segmentation where each pixel is classified as an image. As the final fully connected layer is substituted by a convolutional layer in the FCN, resulting in the superior segmentation effects, it has been extensively studied for the diagnosis of digestive cancers. Oda et al. [51] employed a three-dimensional FCN model to segment the pancreas automatically using CT images and an average Dice score of 89.7 ± 3.8, was obtained. The Dice score indicates the precision of the segmentation model employed by eliminating false positives and is computed as follows:

(1) $$Dicescore=2\times \frac{areaofoverlapbetweentwoimagesets}{totalnumberofpixelsinbothimages}$$

Generally, a Dice score above 88% is considered highly precise. In another study, Guo et al. [52] employed a Gaussian mixture model and used morphological operations on a three-dimensional U-Net segmentation technique, to achieve an improved segmentation accuracy with a Dice score of 83.2 ± 7.8%. It is also evident from the various reports, that the type of AI tool employed will be different for various imaging techniques. The following sections highlight some recent AI-based strategies for different imaging modalities.

## 4. Endoscopic Ultrasound (EUS)

Endoscopic ultrasound (EUS) employs high-frequency ultrasound (US) for the visualization of the size and location of the primary tumor in the pancreas. The ultrasound probe can be maneuvered close to the pancreas for acquiring images of the entire pancreas or the specific locations of suspicious masses or lesions [53]. Advances in the transducer design and the advent of colour Doppler techniques, have contributed to an improved diagnosis and staging of pancreatic cancer. Currently, the sensitivity of EUS, for identifying cancerous lesions in the pancreas, lie in the range 85–99%, that is comparatively superior to CT techniques. Specifically, EUS can detect small lesions in the range of 2–3 mm [54]. For instance, the accuracy of diagnosis for pancreatic tumors with a diameter of 3 cm was reported to be 93% for EUS images, which was significantly superior to CT (53%) and MRI (67%) techniques [55]. Though several literature reports have highlighted the effectiveness of EUS over other medical imaging techniques for the diagnosis of pancreatic cancer and its staging, the resectability has been found to be better predicted only using a combination of CT and EUS images [56,57]. The EUS-driven fine needle aspiration (EUS-FNA) technique has enabled tissue sampling and the evaluation of the primary tumour site, as well as the neighbouring lymph nodes with nearly 100% specificity, that otherwise pose a challenge for detection, using other imaging modalities [58]. The EUS-FNA combination achieved diagnostic accuracies of up to 85%, that are a significant improvement over the 50% accuracy obtained using a CT-assisted diagnosis [59]. However, the EUS-FNA combination is not available in many healthcare institutions. Additionally, the combination requires experienced operators for the precise insertion of the needle that has a major bearing on the diagnostic outcomes [60]. 

One of the major challenges for clinicians is to distinguish cancerous lesions in the presence of chronic pancreatitis (CP), as the neoplastic features are masked by the inflammation [61]. Norton et al. in 2001 [62], employed neural network models to analyse EUS images for differentiating pancreatic ductal adenocarcinoma (PDAC) and CP, using four different image parameters. Though a high sensitivity was achieved, this strategy resulted in a poor specificity of only 50%. In another attempt, Zhu et al. [63] employed a support vector machine model to extract features from EUS images recorded for 262 individuals affected with pancreatic cancer and 126 individuals with CP. The model extracted 105 distinctive features out of which 16 were selected to differentiate pancreatic cancer and CP with a 94% sensitivity. Similarly, the SVM was used by Zhang et al. [40] to differentiate PDAC and normal tissue using29 features identified in EUS images with a sensitivity of 97.98%. In another attempt, Das et al. [64] employed a combination of image analysis and ANNs to demarcate the cancerous zones in EUS images, acquired from individuals affected with pancreatic cancer with a high accuracy of 93%. In another effort employing multilayer perceptron neural networks (MNNs), a type of ANN, Ozkan et al. [65] categorized EUS images of non-malignant and malignant tissues, based upon various age groups of the patients namely, <40 y, 40–60 y, and >60 y. The MNNs employ a visible layer that receives an input that is passed onto inner units that are denoted as hidden layers, as they do not directly receive the input. The final hidden layer turns out the output. The error is calculated, based on the deviations from the expected output and these are used to modify the layers to reduce the error during the training period. In another study [66], both one and two hidden layers were employed that exhibited a 97% accuracy with the training data set and a 95% accuracy with the testing data set for discriminating the malignant and non-malignant samples in the different age categories. The high accuracy was achieved for the data sets that were initially segregated into different age groups when compared to their uncategorized counterparts.

Yet another independent study employed MNNs for identifying pancreatic cancer from images of cell clusters, obtained from individuals using fine needle aspiration (FNA). Post-training, the MNN model was found to match the accuracy of an experienced cytopathologist. Additionally, the MNN model was able to predict accurately even inconclusive images, with 80% sensitivity, clearly demonstrating the promise of this tool for the screening of FNA specimens for pancreatic cancer with a conclusive diagnosis, especially those that are deemed inconclusive by cytopathologists. In an interesting study, a computer-assisted diagnosis (CAD) system was developed to analyse EUS images, using deep learning models (EUS-CAD) to identify PDAC, CP, and a normal pancreas (NP). The training set used 920 EUS images and the testing set used 470 EUS images. The detection efficiency was 92% and 94% in the validation and testing phases, respectively. Errors in diagnosis were identified only using the multivariate analysis of non-PDAC cases that was attributed to mass formation resulting in an over diagnosis of tumours [67].

EUS images of intraductal papillary mucinous neoplasms (IPMNs), that are precursors of PDAC, were analysed using deep learning algorithms to predict malignancy, using EUS images of patients acquired before a pancreatectomy. A total of 3970 images were used for the study and the malignant probability was calculated. The probability of the deep learning algorithm to diagnose malignant IPMN was 0.98 (*p* < 0.001) with a sensitivity, specificity, and accuracy of calculated to be 95.7%, 92.6%, and 94.0%, respectively. The accuracy was significantly superior to the corresponding human diagnosis (56.0%) [68]. A comparison of the literature on pancreatic cancer discrimination from EUS images using AI tools revealed that deep learning and ANN techniques exhibited the greatest accuracy, followed by CNNs and the SVM. However, the literature reports chosen for the study had used images that compared normal and pancreatic cancer while some had tried to differentiate pancreatic cancer with CP. Similarly, the size of the cancerous tissues varied between the studies [69]. Therefore, additional studies are required to address if these differences could reflect in the prediction accuracy of the AI tool employed.

## 5. MRI

MRI is used to visualise the thinned slices of two-dimensional or three-dimensional soft tissues, due to the presence of water molecules in our body. The shift in the precessional frequency and alignment of the nuclei of the protons in the water molecule, in the presence of an external applied magnetic field and radiofrequency, is used for acquiring the image. The technique measures the relaxation times, T1 and T2 that denote the spin-lattice and spin-spin relaxation, respectively, to reach the original equilibrium position [70]. Relaxitivities (r1 and r2), which are the inverse of the respective relaxation times are also measured. Most of the cases employ positive or negative contrast agents, such as gadolinium-based chelates or iron oxide, respectively, to significantly enhance the ratio of the relaxivities for an improved resolution and sensitivity [71].

Early detection of pancreatic cancer is essential to provide the affected individual with a fair chance of survival beyond five years. However, most imaging techniques, including MRI, fail to identify conclusively subtle changes observed in the pre-malignant stages, such as the pancreatic intraepithelial neoplasia, which is commonly associated with the tumorigenesis of PDAC [72]. Even an individual with stage I (localized) pancreatic cancer has only a 39% survival rate over a five-year period [73]. In a typical example of the use of AI for diagnosis using MRI images, a supervised machine learning (ML) algorithm was developed to predict the overall survival rates in PDAC affected patients, using a cohort of 102 MRI images during training and a further 30 images during the testing period [74]. The algorithm was used to segment the images’ extract features. The sensitivity of the ML algorithm was 87%, while the specificity was determined to be 80%. The considerable overlap between the clinical histopathological conclusions and the ML-driven predictions indicates the promise of this strategy for classifying pancreatic cancer sub-types and diagnosis. 

Another study [75] had investigated the ability of deep learning to distinguish between different pancreatic diseases from magnetic resonance (MR) images that were contrast-enhanced, using the T1 contrast agent gadopentetate dimeglumine. The generative adversarial network (GAN) form of machine learning can generate new sets of data which resemble/mimic the data used for training. GAN was employed to generate synthetic images that augmented the T1 contrast enhanced MRI data of 398 subjects within the age range of 16 and 85 years, acquired before the commencement of any treatment from a single hospital centre. The Inception-V4 network, a type of CNN with multiple hidden layers, was trained on the GAN augmented data set. Following the training, the MRI images acquired from two different hospital centres, comprising 50 images from subjects in the age group 24–85 years, and 56 images from patients aged between 26–80 years, were used for validating the performance of the Inception-V4 network towards the disease classification. The results were compared with the predictions made by the radiologist. 

To augment the diagnostic accuracy of MRI on paediatric pancreatic cancer, Zhang et al. [76] used a quantum genetic algorithm to optimize the parameters of a traditional SVM classification model, for the improved prediction accuracy. In addition, this study acquired test samples from real life cases, and assessed the image processing performance of the algorithm for an efficient detection. The results revealed that the model distinguished clearly the cancer features with a high accuracy when compared with the conventional detection algorithm. Another study had employed a robust and intelligent method of ANNs combined with the SVM for the classification of pancreatic cancer to improve the diagnostic process, in terms of both accuracy and time [77]. Here, features of the MR images of the pancreas were extracted using the GLCM (gray-level co-occurrence matrix) method, a second order image texture analysis technique, that defines the spatial relationships among pixels in the region of interest. The best features extracted, using the JAFER algorithm, were analysed using five classification techniques: ANN BP (back propagation ANN), ANN RBF (radial basis function ANN), SVM Linear, SVM Poly (polynomial kernel), and SVM RBF (radial basis function SVM). The two best features selected, using the ANN BP techniques were used for the classification of pancreatic cancer with a 98% accuracy. 

Corral et al. [78] employed a deep learning tool to identify neoplasia in intraductal papillary mucinous neoplasia (IPMN), using CNNs for the classification of the MRI scans of the pancreas. The classification was based on the guidelines issued by the American Gastroenterology Association, as well as the Fukuoka guidelines. When tested in 139 MRI scans of individuals, among which 22% were of a normal pancreas, 34% had a low-grade dysplasia while 14% were diagnosed with a high-grade dysplasia and the remaining 29% had adenocarcinoma, the model exhibited a detection sensitivity of 92% and a specificity of 52% for the detection of dysplasia. The deep learning technique exhibited an accuracy of 78%, in comparison to the 76% obtained by the classification using the American Gastroenterology Association guidelines. 

For improving the accuracy, reliability, and efficiency of diagnosis, Chen et al. [79] developed an automated deep learning model (ALAMO) for the segmentation of multiple organs-at-risk (OARs) from the clinical MR images of the abdomen. The model had included training procedures, such as Multiview, deep connection, and auxiliary supervision. The model used multislice MR images as the input and generated segmented images as the output. The model was investigated using ten different OARs, such as the pancreas, liver, spleen, stomach, duodenum, small intestine, kidneys, spinal cord, and vertebral bodies. The results from the model correlated well with those obtained using the manual techniques. However, further studies integrating AI-based algorithms with these ALAMO generated segmented MR images of the pancreas are required for the extraction of features to confirm the onset or progression of PC.

## 6. Computed Tomography

A computed tomography (CT) scan is a non-invasive clinical imaging technique that employs X-rays to obtain images at different angles. The resultant images are processed using customized software to obtain a reconstructed 3D image, which provides valuable anatomical information [80]. This technique is widely employed in healthcare centres for the diagnosis of tumours or internal injuries [81,82]. Despite its merits, CT scan images pose a challenge to clinicians for the accurate diagnosis of cancers, owing to irregular contours presented by regions with lesions, vasculature, bony structures, and soft tissues that display a mosaic of densities and intensities [83]. Additional challenges involved in the precise prediction of the disease from the CT scans are associated with fuzzy and noisy images that lack adequate contrast [84]. AI-driven methods that enable image segmentation, contour identification, and disease classification, therefore will be invaluable in improving the prediction efficiency for pancreatic diseases from CT images [85]. The currently employed conventional image segmentation models consume considerable computational time and power, as they perform every operation for each pixel in the image [86]. Further, the resultant processed image quality also lacks quality, thereby necessitating the development of more robust tools for AI-driven tools for image segmentation and processing that may provide a better diagnostic accuracy [87]. In an interesting study [88], about 19,500 non-contrast CT scan images, acquired from 469 scans, were segmented using CNNs and the mean pancreatic tissue density, in terms of the Hounsfield unit (HU), as well as the pancreatic volume, were computed using the CNN algorithm. The comparison of the results of the pre-diagnostic scans from individuals who later developed PDAC and those that remained cancer-free, revealed that there was a significant reduction in the mean whole gland pancreatic HU of 0.2 vs. 7.8 in individuals who developed PDAC. This suggests that the attenuation of the HU intensity in the CT images of the pancreas could imply a risk of PDAC. This study has opened new avenues for employing CNNs as a tool for the pre-diagnosis/very early diagnosis of PDAC from CT scan images.

In another attempt to classify PDAC, a regular CNN algorithm with four hidden layers was trained using CT images obtained from 222 affected individuals and 190 non-cancerous individuals. Though a diagnostic accuracy of 95% was achieved using CNNs, it was not superior to the predictions made by human experts indicating the need for an appropriate AI architecture for the classification of pancreatic cancer [89]. Zhang et al. [90] employed feature pyramid networks with a recurrent CNN (R-CNN) that could identify the sequential patterns and predict the subsequent patterns of a given data set for classifying PDAC from CT scan images. A dataset of 2890 CT images was employed for training the network to achieve a classification accuracy of about 94.5%. Though this method proved to be superior to the existing methods, it was limited by the input uncertainty that is generally associated with closed-source data. This drawback could be eliminated by using a public data set for training. In a more advanced variant, a 16-layer VGG16 CNN model was employed along with R-CNN to diagnose PDAC from 6084 enhanced CT scans obtained from 338 PDAC-affected individuals. The combination of VGG16 and R-CNN exhibited a high prediction accuracy of about 96%. Each CT image was processed by the R-CNN within 0.2 s that was considerably faster than a clinical imaging expert [6]. Additionally, a deep learning algorithm has been developed by Chen et al. [91] for detecting pancreatic cancer that is smaller than 2 cm on CT scans. The study result showed that the CNN was effective in distinguishing patients with pancreatic cancer from normal pancreatic individuals, achieving an 89.7% sensitivity and a 92.8% specificity. It also showed a higher sensitivity of 74.7% for the identification of pancreatic cancer malignancies, smaller than 2 cm.

An attempt to employ CNN models to distinguish different kinds of pancreatic cysts was made using CT images from 206 patients. Among these individuals, 64 suffered from intraductal papillary mucinous neoplasms (IPMNs), 66 had been diagnosed with serous cystic neoplasms (SCN), 35 had mucinous cystic neoplasms (MCNs) while 41 individuals suffered from solid pseudopapillary epithelial neoplasms (SPENs). The feature extraction from the CT images and classification of the type of pancreatic cyst, was accomplished using densely connected convolutional network (Dense-Net) architecture that uses dense layers which receives inputs from all neurons/nodes and dense blocks connecting all layers by the shortest route. The Dense-Net algorithm performed better than the conventional CNN model in discriminating between the different types of cysts with the highest accuracy of 81.3% observed for IPMNs followed by 75.8% for the SCNs and 61% for the SPENs [92]. Though the Dense-Net model outperformed the CNNs in all categories, the study lacked information on the tumour size and failed to provide reasons for the positive and negative errors encountered in the identification of the type of pancreatic cysts. The model needs to be tested rigorously with a wider range of cysts to understand its capability for discriminating between different types of pancreatic cysts if it is to be adopted in the clinics. 

## 7. Positron Emission Tomography (PET)

Positron emission tomography (PET) employs short-lived radioisotope tracers that emit positrons. These positrons destructively interact with an electron to generate photons, which are recorded for generating the PET image. The tracer can be differentially localized in various tissues by conjugating with a biomolecule for a better target specificity [93,94]. The PET scans provide additional information about the functioning of an organ. Commonly employed tracers include ^18^F, ^15^O, ^13^N, and ^11^C, that have half-lives of 109.74 min, 122.24 s, 9.97 min, and 20.38 min, respectively [95]. PET imaging has been also used to diagnose the recurrence of pancreatic cancer as well as to understand the response of the cancer tissue to different therapeutic interventions. Despite several studies that have shown the diagnostic efficiency of PET scans towards pancreatic cancers with a sensitivity in the range of 85% and above [96], several factors, such as the dysregulated glucose metabolism and inflammation interfere with the sensitivity of the diagnosis from PET images, resulting in false positives [97]. PET scans are also ineffective in diagnosing pancreatic cancers when the tumour mass has a diameter below 2 cm [98]. This necessitates the use of advanced AI-aided algorithms for the discrimination and classification of cancerous masses from the PET scan images.

For imaging cancers, ^18^F substituted glucose or fluorodeoxyglucose (FDG) has been frequently used, due to the high consumption of glucose by cancer cells to meet its metabolic requirements [97]. PET scans have been employed frequently in conjunction with MRI or non-contrast CT, owing to their poor spatial resolution for the diagnosis of cancers, including their staging [99]. To overcome challenges in discriminating cancerous lesions from non-contrast CT images, 18F- FDG PET/CT imaging of pancreatic cancers was used by Li et al. [100], in conjunction with a SVM algorithm. The region of interest (ROI) identified in the CT image of the pancreas was initially segmented, using a simple linear iterative clustering (SLIC) followed by the feature extraction using the dual threshold principal component analysis (DT-PCA). Finally, a hybrid feedback-SVM-random forest algorithm (HFP-SVM-RF) was used to classify the pancreatic cancerous lesions. The random forest model is a type of supervised machine learning model that is widely used for classification and decision making. The hybrid model exhibited an accuracy of 96.5% when tested using the PET/CT images of 40 patients with pancreatic cancer and 40 non-cancer individuals. The hybrid algorithm when tested using 82 public PET/CT scans exhibited a similarity score of 78.9% and 65.4%, when compared with the ground-truth contours using the Dice coefficient and Jaccard index, respectively, suggesting there is scope for further improvement in the diagnostic performance.

Radiomics is a feature extraction method that has been widely used in image processing tools. A combination of radiomics with machine learning was employed for the prognostic prediction of the survival rate from ^18^F-FDG-PET scans of 138 patients with pancreatic cancer. A random forest model was used for the classification of 42 features extracted from the PET images. The model revealed that the gray-level zone length matrix (GLZLM), the gray-level non-uniformity (GLNU) in the images as the top factor that influenced the one year survival, while the total lesion glycolysis ranked second. This information was used to stratify individuals into poor prognosis groups with a high risk of mortality [101]. 

It is thus evident that every imaging technique will require customized robust algorithms to extract the subtle but distinctive features of pancreatic cancer for the accurate identification and stratification. The evolution of new ML algorithms continues to improve the sensitivity and selectivity of the diagnosis of pancreatic cancer at an early stage, thereby improving the survival chances of the affected individual. Table 2 lists some of the major studies, using various AI driven models for the diagnosis of pancreatic cancer.

## 8. Pancreatic Cancer Risk Prediction Using AI

Since pancreatic cancer is a highly aggressive form of cancer that is largely asymptomatic in the early stages and has a tendency to spread rapidly, leading to poor survival duration post-diagnosis, the AI-based prediction of the risk of developing pancreatic cancer could be an immensely useful strategy for improving the prognosis for an individual. Muhammad et al. [112] had successfully employed ANNs from personal health data to predict and stratify the pancreatic cancer risk as a low, medium, or high risk ,with a sensitivity and specificity of 80.7%.This study highlights the ability of the AI-based predictive tools for the effective management of the pancreatic cancer risk even before the manifestation of symptoms. Similarly, Corral et al. [78] had employed an AI algorithm to identify pancreatic cysts that pose a high risk of transforming into cancerous lesions. Such a pre-diagnosis could help clinicians in designing adequate preventive interventions to save lives. The detection of subtle textural and morphological changes in CT and MRI scans of the pancreas could also be facilitated through customized AI algorithms [117]. Several attempts have also been reported to employ AI tools to predict the risk of developing pancreatic cancer from biomarker measurements, as well as abdominal scans to discern pre-cancerous abnormalities [117].

## 9. AI-Driven Diagnosis Based on Cancer Biomarkers

The serological detection of PC is based on the quantification of a biomarker whose levels are altered in cancerous conditions. However, a single marker could not accurately diagnose a specific type of cancer as there are several other conditions that could modulate the levels of said biomarker. Hence, multiple biomarkers need to be analysed, to accurately diagnose PC. In an earlier work, protein markers from the serum of 27 normal and 27 individuals diagnosed with pancreatic cancer, were profiled using surface-enhanced laser desorption ionization (SELDI), and were classified using a decision tree algorithm, based on which six serum proteins were identified as pancreatic cancer biomarkers [118]. Carbohydrate antigen 19-9(CA19-9) is the most extensively explored protein biomarker of pancreatic cancer. However, several studies have indicated that CA19-9, by itself, could not be an effective predictor of pancreatic cancer and hence the search for additional diagnostic protein markers in serum are underway [119]. Analysis of datasets from microarray and the next generation sequencing of samples for the gene expression or serum protein expressions using deep learning and machine learning algorithms, could aid in identifying the most promising protein biomarkers that aid in the early detection of pancreatic cancer. For instance, the SVM based algorithm, in combination with the recursive feature elimination (RFE), was employed to screen the gene expression datasets of 78 samples, for additional pancreatic cancer biomarkers. Seven gene targets were short-listed among the genes encoding for the proteins FOS that encodes for the leucine zipper protein, MMP7 (matrix metalloproteinase-7), and A2M (alpha-2-macroglobulin), were predicted to be more accurate diagnostic markers for pancreatic cancer, not only in serum, but also in urine samples [120]. Similarly, ANN-based methods have been employed to analyse the levels of key serum biomarkers implicated in PC, such as CA19-9, CA125, and carcinoembryonic antigen (CEA), from 913 samples obtained from individuals with a normal and a cancerous pancreas. The results showed an improved detection accuracy when compared with a single marker-based prediction, clearly highlighting the benefits of an AI-integrated multi-analyte diagnosis [121]. Exosomes, which are vesicular structures containing miRNA, specific to the source cells, are gaining importance for the disease diagnosis. Several exosome entrapped miRNA have been identified in PC, such as miR-16, miR-20a, miR-21, miR-21-5p, miR-24, miR25, miR99a, miR-133a, miR185, miR191, miR-196a, miR-223, miR-642b-3p, miR-663a, miR-1290, miR-1246, miR-5100, and miR-8073 [122]. In a seminal work, the exosomes obtained from a panel of mouse and human origin PC cell lines, were captured using antibodies against the surface expressed EpCAM (epithelial cell adhesion molecule). The RNA cargo was isolated from the exosomes and the miRNA was identified using qPCR. The cancer miRNA signatures were identified using a custom-developed machine learning algorithm. The system was validated using samples isolated from individuals with a normal pancreas and those with pancreatic cancer, with a good prediction accuracy [123]. In another study, a neural network algorithm was employed to screen 140 datasets of individuals diagnosed with pancreatic cancer, for gene biomarkers in urine samples, namely REG1A/1B, LYVE1, TFF1, and CA19-9. Following the training, the neural network algorithm predicted REG1A/1B as the most important biomarker in the urine samples with an importance ratio exceeding 80% [124]. With the discovery of new circulating markers, such as glycoproteins and genetic markers, such a machine learning-based diagnosis could herald in the rapid and accurate detection of PC.

The histological analysis or tissue biopsies have been conventionally employed for the identification and stratification of cancers. However, this is a time-consuming process. Further, there is a constant increase in the number of samples that are sent for analysis to the anatomical pathological laboratory and this, coupled with insufficient skilled pathologists, leads to long turn-around-times [125]. Additionally, cytopathology requires the accurate slide preparation and optimal staining of the tissue slices. However, the staining intensity of biopsy slides exhibit analyst-based, sample thickness-based and laboratory protocol-based variations in the intensity [125]. In this context, deep learning algorithms, such as VGG, DenseNet, ResNet etc., and machine learning algorithms, based on SVM and the random forest, can be employed to extract specific tumour features from the tissue slices to improve the speed of detection and reduce the burden on the clinical pathologists. Similarly, the use of algorithms, such as SA-GAN (stain acclimatization generative adversarial network) that employs a generator that imports the input source image and generates a target image that incorporates the features of the input sample and the colour intensity of a training sample. Two discriminators are also incorporated into this deep learning model, which ensure that the colour intensity of the desired training image and textural features of the source image are maintained in the generated image, thus ensuring the stain colour normalization across the different images [126]. Such approaches have been attempted, to identify various types of gastrointestinal and breast cancer, using mammograms and tissue biopsies [127]. Using a similar concept, a deep learning-based spiral algorithm was employed to transform 3D MRI images of the pancreatic tissue into 2D images without compromising then original image texture and edge parameters. The CNN-based models were employed for the feature extraction and the bilinear pooling module was used to improve the prediction accuracy. Parameters, such as size, shape, volume, texture, and intensity, were employed to classify the image as pancreatic cancer with TP53 gene mutation or otherwise. The prediction results agreed well with the actual mutation status. This approach overcomes the drawback of the need for painful biopsies for classifying a tumour as TP53 positive. In addition, this novel method offers a non-invasive approach for predicting gene mutations, using AI-driven cytopathology that may also be extended for other forms of cancer or gene mutations [128]. Similarly, ResNet and DenseNet models have been employed to identify *Helicobacter pylori*, a key causative pathogen in different gastric cancers from stained tissue biopsy specimens [129]. The advantage of using machine learning models in this case over conventional cytopathology, is the ability of the model to identify even small numbers of the bacteria, which is very tedious and time-consuming in the conventional mode. Abnormal goblet cells have been identified with an 86% accuracy in tissue samples of individuals with Barrett’s esophagus, using VGG algorithms [130]. AI-driven algorithms can be useful in detecting microsatellite instabilities in the biopsy samples, that area hallmark of many forms of cancers [125]. These studies clearly demonstrate that the integration of machine learning in cytopathology can be useful for the faster, efficient, and early diagnosis of pancreatic cancer. This field is slowly gaining prominence and may soon lead to the establishment of a digital cytopathology as a mainstay in the detection and stratification of cancers.

## 10. Ethics of Using AI for Diagnosis

Though AI offers a plethora of benefits in improving the detection and stratification of PC, there are several ethical concerns that have emerged among a section of the society, on the extensive use of AI-based diagnostics. Since AI tools require large datasets for training and validation, concerns on data privacy and confidentiality have been raised. Additionally, data security and safety issues have also been associated with use of an AI-based diagnosis [131]. There exists a regulatory vacuum in the realm of AI-based tool development and no structured white document is available on the data collection, storage, processing and sharing. Furthermore, frequent comparisons between expert predictions by clinicians and the AI algorithm, have given rise to the theory of inadequate training or de-skilling of clinicians, in future, owing to the over-dependence of AI-based detections. A lack of patient-doctor connect, or dissolution of the trust factor are additional issues that have been associated with the deployment of AI-driven technologies in healthcare [132]. Accountability and professional responsibility issues, in the case of a wrong diagnosis by AI-based tools, that may result in disastrous consequences, is another facet that is being debated as a negative aspect of all AI-driven cancer diagnoses.

## 11. Concluding Remarks

The use of AI for cancer detection and biomarker discovery, is expected to be the target of several research studies involving AI over the next decade. Several studies in this direction have clearly demonstrated the benefits of the AI-driven detection of pancreatic cancer, especially those employing imaging tools. However, the widespread clinical deployment of this technology is yet to be realized, owing to lack of large datasets to convincingly train and validate the developed algorithms. Most AI-based models have been developed in a ‘black box’ mode and as a result, the clinicians are unable to understand or explain the basis of identification or stratification, thereby leading to a reticence in employing this technology. Additional ethical issues concerning data privacy and security further have slowed down the translation of an AI-based diagnosis in clinics. However, the exponential growth, witnessed in computing resources, including open-source tools, has triggered an avalanche of studies focused on developing more robust algorithms for the accurate, rapid, and early diagnosis of PC. As this field continues to grow, new regulatory policies concerning its use and deployment will emerge so that the benefits of this technology can be harnessed to save lives.

## Figures and Tables

**Table 1:** Major imaging techniques employed for the diagnosis of pancreatic cancer and their limitations.

Technique	Merit(s)	Demerit(s)
Multidetector computed tomography (MDCT)	High sensitivity and specificity for the detection of the vascular invasion; Short acquisition time; 3D image processing aids in the staging of the cancer; Obtaining thin collimation images with a high spatial and temporal resolution.	Nephrotoxicity; Tissue/organ damage due to the radiation exposure; Lack of an attenuation gradient between the cancer tissue and pancreatic parenchyma, leading to erroneous predictions.
Magnetic resonance imaging (MRI)	Low risk of ionizing radiation; Better sensitivity, specificity, and accuracy when compared to CT techniques; Non-invasive imaging of the pancreato-biliary system by magnetic resonance cholangio-pancreatography (MRCP).	Expensive; Limited availability; Problems associated with individuals having metal implants.
Endoscopic ultrasound (EUS) with or without fine needle aspiration (FNA)	Can detect small cancerous lesions 2–5 mm in dimension; Highest diagnostic accuracy; Highly specific; Loco-regional staging can be detected.	Cannot detect extra-abdominal metastasis; Limited availability; Requires a trained operator.
Positron emission tomography (PET)	Useful in detecting metastasis.	Staging of pancreatic cancer cannot be conclusively determined; Expensive; Exposure to radiation.

**Table 2:** Summary of the AI driven models for the pancreatic cancer diagnosis.

Modality	AI Model	Study Population	Purpose	Sensitivity	Specificity	Accuracy	Reference
CT	CNN	27	Pancreatic cystic neoplasm malignancy prediction	-	-	92.9	Watson et al., 2021 [102]
CT	Naïve Bayer classifier	72	PDAC identification	-	-	86	Ahamed et al., 2022 [103]
CT	CNN	1006	Pancreas segmentation	-	-	-	Lim et al., 2022 [104]
CT	CNN	68	Serum tumor marker analysis	89.31	92.31	-	Qiao et al., 2022 [105]
CT	CNN	513	Pancreatico enteric Anastomotic Fistulas prediction after a pancreatoduodenectomy	86.7	87.3	87.1	Mu et al., 2020 [106]
CT	ANN	62	Acute pancreatitis risk prediction	-	-	-	Keogan et al., 2002 [107]
CT	Support vector machine	56	PDAC histopathological grade discrimination	78	95	86	Qiu et al., 2019 [108]
CT	CNN	370 patients, 320 controls	PC detection	97.3 (Test set 1) 99 (Test set 2)	100 (Test set 1) 98.9 (Test set 2)	98.6(Test set 1) 98.9 (Test set 2)	Liu et al., 2020 [109]
CT	Deep learning	750 patients 575 controls	PDAC detection	-	-	87.8	Chu et al., 2019 [110]
CT	CNN	222 patients 190 controls	PC diagnosis	91.58	98.27	95.47	Ma et al., 2020 [89]
CT	DCNN	2890 CT images	Pancreatic cancer detection	83.76	91.79	94	Zhang et al., 2020 [90]
CT	Deep learning	319	Preoperative pancreatic cancer diagnosis	86.8	69.5	87.1	Si et al., 2021 [111]
CT	ANN	898	Cancer risk prediction	80.7	80.7	-	Muhammad et al., 2019 [112]
CT	CNN	669 patients 804 controls	PC differentiation	89.7	92.8	-	Chen et al., 2022 [91]
MRI	CNN	139	Identification of intraductal papillary mucinous neoplasia	75	78	-	Juan et al., 2019 [78]
MRI	CNN	27	Automatic image segmentation	-	-	-	Liang et al., 2020 [113]
MRI	ANN	168	PDAC differentiation	-	-	96	Devi et al., 2018 [114]
EUS	CNN	583	Autoimmune pancreatitis from PDAC	90	85	-	Marya et al., 2021 [115]
EUS	CAD	920 (Validation) +470 (test)	PDAC detection	-	-	-	Tonozuka et al., 2021 [67]
EUS	ANN	202 (cancerous) & 130 (Non-cancerous)	Computer-aided pancreatic cancer diagnosis using image processing	83.3	93.3	87.5	Ozkan et al., 2019 [65]
EUS	ANN	258	Pancreatic lesion characterization	-	-	91	Saftoiu et al., 2012 [34]
EUS	ANN	388	PDAC and CP differentiation	96	93	94	Zhu et al., 2013 [63]
EUS	ANN	167	PDAC and CP differentiation	94	94	-	Saftoiu et al., 2015 [116]
EUS	ANN	56	Normal, CP and PDAC differentiation	-	-	93	Das et al., 2008 [64]
EUS	ANN	21	PDAC and CP differentiation	-	-	89	Norton et al., 2001 [62]
PET/CT	SVM	80	Pancreatic cancer segmentation	95.23	97.51	96.47	Li et al., 2018 [100]
